# Supplementary material for: Super-Resonant Intracavity Coherent Absorption
Source: Sci Rep. 2016 Jul 1;6:28947. doi: 10.1038/srep28947 (PMC4929439; doi:10.1038/srep28947)
Supplement: Supplementary Information [file srep28947-s1.pdf]

Supplemental material for:

Super-Resonant Intracavity Coherent Absorption

P. Malara[1*], C. E. Campanella[2], A. Giorgini[1], S. Avino[1], P. De Natale[3] and G. Gagliardi[1]

[1] Consiglio Nazionale delle Ricerche, Istituto Nazionale di Ottica (INO), via Campi Flegrei, 34, Comprensorio A. Olivetti, Pozzuoli, (NA), Italy.

[2] QOpSys s.r.l.s. , via Matteotti, 23, Gioia del Colle, Bari, Italy.

[2]Consiglio Nazionale delle Ricerche, Istituto Nazionale di Ottica (INO), Largo E. Fermi, 6, Firenze, Italy

**Corresponding author: pietro.malara@ino.it*

In this supplemental materials we report the detailed derivations of equations 3a and 3b of the main text, describing the effective interaction pathlength of the symmetric and antisymmetric modes of a super-resonant coherent perfect absorber (RCPA). Additional experimental details and data are also provided.

In this section we work out the sensitivity of the symmetric and antisymmetric modes of the RCPA modes to an intra-FP absorption in terms of equivalent absorption pathlength. For simplicity, we consider an internal FP with identical, lossless mirrors of reflectivity R and transmissivity T=1-R, and a refractive index $n_{fp}<n_{ring}$ (the optical field gains a π phase factor only upon reflection towards the ring). In these conditions, the fields reflected and transmitted by the FP are:

$$r_{FP}(\beta) = -\sqrt{R}\left(1 - \frac{Td^2e^{2i\beta l}}{1 - e^{2i\beta l}Rd^2}\right)\ ,\quad t_{FP}(\beta) = \frac{Tde^{i\beta l}}{1 - e^{2i\beta l}Rd^2} \tag{S0}$$

Where β is the optical wavenumber, d is the per-pass field intracavity transmission of the FP and l its length. In the close vicinity of a resonance, where $\beta l \sim \pi$, the above expressions become

$$r_{FP} = -\sqrt{R}\left(1 - \frac{Td^2}{1 - Rd^2}\right), \qquad t_{FP} = -\frac{Td}{1 - Rd^2} \tag{S1}$$

Equation S1 can be used to calculate $|t_{FP} + r_{FP}|$ and $|t_{FP} - r_{FP}|$. After some algebraic passages, we get:

$$|t_{FP} + r_{FP}| = \left|\frac{\sqrt{R} + d}{1 + \sqrt{R}d}\right| \tag{S2a}$$

$$|t_{FP} - r_{FP}| = \left|\frac{d - \sqrt{R}}{1 - \sqrt{R}d}\right| \tag{S2b}$$

In the presence of an absorbing sample (with absorption coefficient α) homogeneously distributed over the internal length of the FP resonator, we can consider $d = e^{-\frac{\alpha}{2}l}$. For small absorption ($\alpha l \rightarrow 0$), $e^{-\frac{\alpha}{2}l} \sim 1 - \frac{\alpha l}{2}$. Substituting *d* in Eqs. (S2):

$$|t_{FP} + r_{FP}| = \frac{1 + \sqrt{R} - \frac{\alpha}{2} l}{1 + \sqrt{R} - \sqrt{R}\frac{\alpha}{2} l} \qquad (S3a)$$

$$|t_{FP} - r_{FP}| = \frac{1 - \sqrt{R} - \frac{\alpha}{2} l}{1 - \sqrt{R} + \sqrt{R}\frac{\alpha}{2} l} \qquad (S3b)$$

By dividing numerator and denominator by *1-R* we obtain:

$$|t_{FP} + r_{FP}| = \frac{\frac{1}{1-\sqrt{R}} - \frac{\alpha l}{2(1-R)}}{\frac{1}{1-\sqrt{R}} - \frac{\sqrt{R}}{2}\frac{\alpha l}{1-R}} \qquad (S4a)$$

$$|t_{FP} - r_{FP}| = \frac{\frac{1}{1+\sqrt{R}} - \frac{\alpha l}{2(1-R)}}{\frac{1}{1+\sqrt{R}} + \frac{\sqrt{R}}{2}\frac{\alpha l}{1-R}} \qquad (S4b)$$

At this point, we introduce the assumption that the single-pass absorption in the FP is negligible compared to the FP outcoupling: $\frac{\alpha l}{1-R} \sim 0$. In this approximation, we consider $|t_{FP} + r_{FP}|$ and $|t_{FP} - r_{FP}|$ as functions of $\frac{\alpha l}{1-R}$ and linearize them around 0, obtaining

$$|t_{FP} + r_{FP}| = 1 + \frac{1}{2}\sqrt{R}\left(\frac{1-\sqrt{R}}{1+\sqrt{R}}\right)\alpha l \qquad (S5a)$$

$$|t_{FP} - r_{FP}| = 1 - \frac{1}{2}\sqrt{R}\left(\frac{1+\sqrt{R}}{1-\sqrt{R}}\right)\alpha l \qquad (S5b)$$

As a final remark, we recall that the finesse of a Fabry-Perot is given by $\mathcal{F}_{FP} = \frac{\pi\sqrt{R}}{1-R}$, and that $\frac{1+\sqrt{R}}{1-\sqrt{R}} \sim 4\frac{\mathcal{F}_{FP}}{\pi}$, as shown in fig.S1. With this in mind, Eqs (S5) can be written as

$$|t_{FP} + r_{FP}| = 1 + \frac{1}{8}\sqrt{R}\left(\frac{\mathcal{F}_{FP}}{\pi}\right)^{-1}\alpha l \qquad (S6a)$$

$$|t_{FP} - r_{FP}| = 1 - 2\sqrt{R}\frac{\mathcal{F}_{FP}}{\pi}\alpha l \qquad (S6b)$$

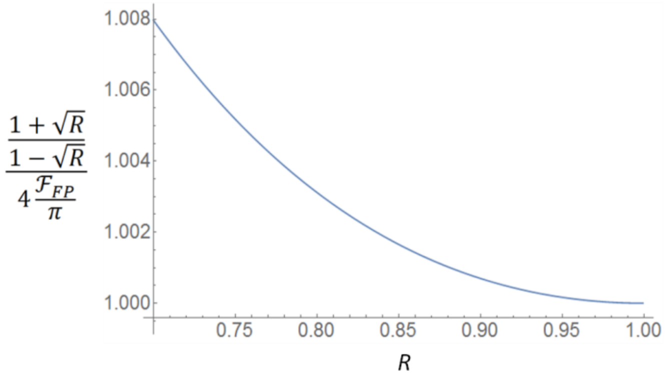

Fig.S1: validity of the approximation used in Eqs.(S6).

We can write the RCPA transmission by substituting t_{FP} and r_{FP} in equation (1) of the main text:

$$\frac{E_{out}}{E_{in}} = -\frac{1}{2}\left(\frac{k^2|t_{FP}+r_{FP}|e^{-i\left[\frac{\beta L}{2}+\arg(t_{FP}+r_{FP})\right]}}{1-\tau^2|t_{FP}+r_{FP}|e^{-i[\beta L+\arg(t_{FP}+r_{FP})]}} + \frac{k^2|t_{FP}-r_{FP}|e^{-i\left[\frac{\beta L}{2}+\arg(t_{FP}-r_{FP})\right]}}{1-\tau^2|t_{FP}-r_{FP}|e^{-i[\beta L+\arg(t_{FP}-r_{FP})]}}\right)$$

k and τ are the coupling coefficients of the ring optical couplers (assumed identical). For separated peaks (no interference), the intensity of the symmetric and antisymmetric resonances, normalized to their peak transmission I_0 (in absence of absorption) is:

$$\frac{I_{sym}}{I_0} = \frac{K^2|t_{FP}+r_{FP}|^2}{(1-\Gamma|t_{FP}+r_{FP}|)^2} \qquad (S7a)$$

$$\frac{I_{asym}}{I_0} = \frac{K^2|t_{FP}-r_{FP}|^2}{(1-\Gamma|t_{FP}-r_{FP}|)^2} \qquad (S7b)$$

Where $\Gamma = \tau^2$ and $K = k^2$. We note that the above expressions are analogous to the normalized transmissions of two independent rings with per-pass field intracavity transmission coefficient $d_{eff} = |t_{FP} \pm r_{FP}|$ respectively.

We now substitute in equations (S7) the $|t_{FP} \pm r_{FP}|$ calculated in equation (S6). For ease of notation, we use a generic expressions $1 + \mathrm{A}^{\pm}\alpha l$. We will distinguish symmetric and antisymmetric transmission at the end of the calculation, by substituting $\mathrm{A}^{\pm}$ with the coefficients of αl of equation (S6).

$$\frac{I}{I_0} = \frac{K^2(1+(\mathrm{A}^{\pm}\alpha l)^2 + 2\mathrm{A}^{\pm}\alpha l)}{1+\Gamma^2+\Gamma^2(\mathrm{A}^{\pm}\alpha l)^2 - 2\Gamma - 2\Gamma\mathrm{A}^{\pm}\alpha l} \qquad (S8)$$

Neglecting the quadratic terms in αl we rearrange in

$$\frac{I_{out}}{I_0} = \frac{K^2}{(1-\Gamma)^2 - 2\Gamma\mathrm{A}^{\pm}\alpha l} + \frac{2K^2\mathrm{A}^{\pm}\alpha l}{(1-\Gamma)^2 - 2\Gamma\mathrm{A}^{\pm}\alpha l} \qquad (S9)$$

Again the second term on the right-end side is negligible for $\alpha l \to 0$.

$$\frac{I_{out}}{I_0} = \left(\frac{K}{1-\Gamma}\right)^2 \frac{1}{1 - \frac{2\Gamma}{1-\Gamma} \mathrm{A}^{\pm} \alpha l} \qquad (S10)$$

Then, we get to the absorbance:

$$\frac{\Delta I^{\pm}}{I_0} = 1 - \frac{1}{1 - 2\Gamma \mathrm{A}^{\pm} \frac{\alpha l}{1-\Gamma}} \qquad (S11)$$

Analogously to what done previously for the internal FP cavity, if the single-pass absorption is small also compared to the coupling losses of the ring resonator $\left(\frac{\alpha l}{1-\Gamma} \sim 0\right)$, we can linearize the absorbance (S11) obtaining

$$\frac{\Delta I^{\pm}}{I_0} = 2\Gamma \mathrm{A}^{\pm} \frac{\alpha l}{1-\Gamma} \qquad (S12)$$

At this point we can finally write the absorbance of the symmetric and antisymmetric modes by substituting the coefficients $\mathrm{A}^{\pm}$ with the coefficient of equation (S6):

$$\frac{\Delta I_{sym}}{I_0} = \sqrt{\Gamma \mathrm{R}} \frac{1}{4} \frac{\mathcal{F}_{\mathrm{ring}}}{\mathcal{F}_{FP}} \alpha l \qquad (S13a)$$

$$\frac{\Delta I_{asym}}{I_0} = \sqrt{\Gamma \mathrm{R}} \frac{4}{\pi^2} \mathcal{F}_{\mathrm{ring}} \mathcal{F}_{FP} \alpha l \qquad (S13b)$$

Now, considering that the absorbance is defined as $\frac{\Delta I}{I_0} = \alpha l_{eff}$, we get the following expression for the effective pathlength of the symmetric and the antisymmetric modes:

$$l_{eff}^{sym} = \sqrt{\Gamma \mathrm{R}} \frac{1}{4} \frac{\mathcal{F}_{\mathrm{ring}}}{\mathcal{F}_{FP}} l \qquad (S14a)$$

$$l_{eff}^{asym} = \sqrt{\Gamma \mathrm{R}} \frac{4}{\pi^2} \mathcal{F}_{\mathrm{ring}} \mathcal{F}_{FP} l \qquad (S14b)$$

Eqs.(S14) (corresponding to Eq.(2) of the main text) state that the pathlength enhancement in the antisymmetric mode scales with the product of the ring and the FP enhancement factors, and is therefore much larger than the traditional resonant enhancement. For the symmetric mode instead, the pathlength enhancement scales as the ratio of the ring and FP enhancement factors, and can therefore be even smaller than 1, which means that the effective absorption of the symmetric mode is smaller than the single-pass absorption.

The situation is illustrated in fig. S2. In the top layer, the modal absorption of a RCFP and the equivalent FPs are plotted for different values of the FP intracavity loss. In the bottom layer, the symmetric and antisymmetric mode absorbance is plotted for a fixed $\mathcal{F}_{FP}$ and different values of $\mathcal{F}_{ring}$, along with the absorbance of the uncoupled FP mode and the single-pass absorbance (dashed black lines).

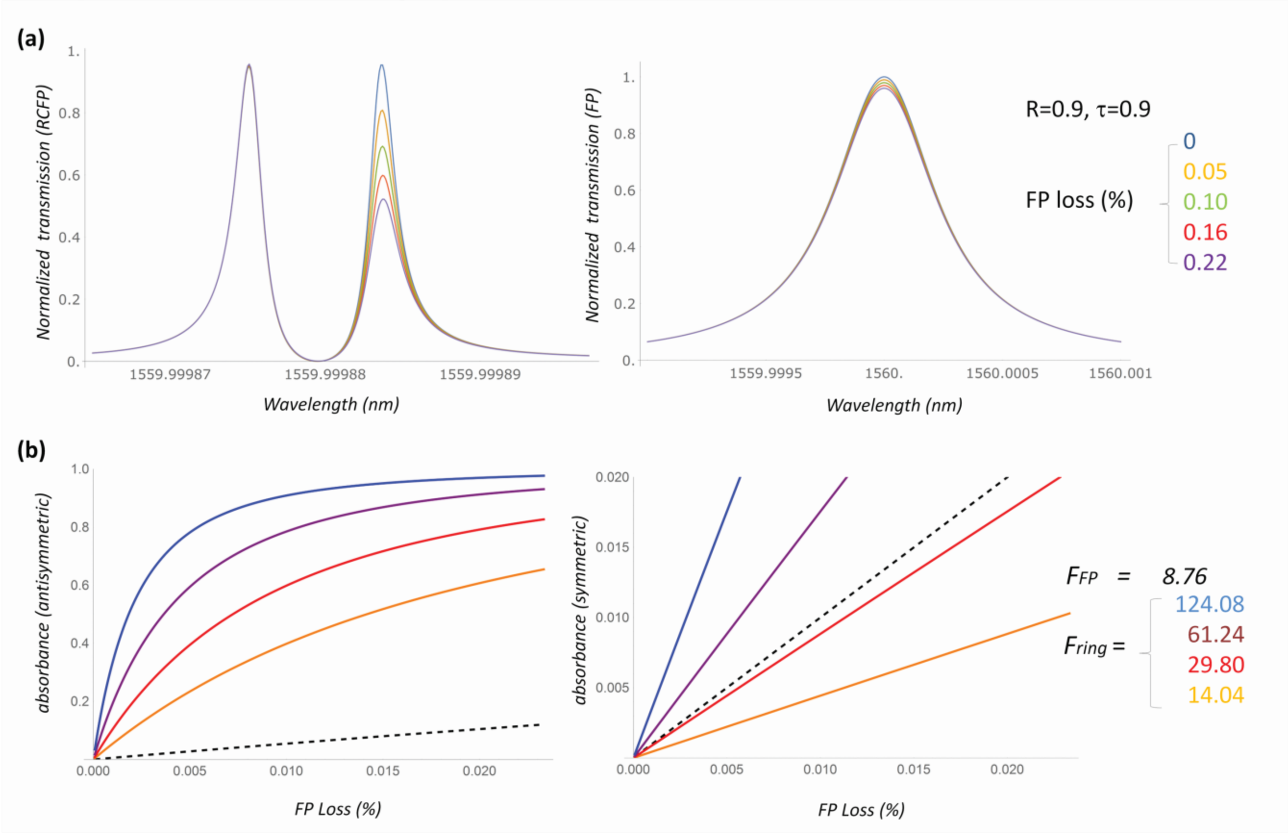

Fig.S2 –weak-absorption behavior of the RCPA modes. **(a)** Resonant modes of the RCPA (left) and the internal FP alone (without ring-coupling) compared for different values of the intracavity loss. **(b)** Absorbance curves of the RCPA dark (left) and bright (right) modes calculated for different values of the ring resonator finesse. The absorbance of the internal FP alone is plotted in dotted line for comparison.

2 – Absorbance measurements.

In these measurements, in an attempt to reduce the saturation of the antisymmetric absorption at the center of the spectrum, the finesse of the internal FP resonator was also reduced from the F_{FP}~25 to F_{FP}~6 by tuning the reflectivity of the cavity FBG-mirrors at the laser wavelength to R=0.6.

For 4 different loss levels, the coupled-resonator transmission and the open-loop Fabry-Perot transmission were acquired. The recorded spectra are shown in Fig. S3. By fitting these data as described in the main text, and normalizing to the same I_0 level, Fig.3a of the main text was obtained. The best fit parameters are shown in table S1. In table S2 instead the best fit parameters for the absorbance curves calculated in the central and lateral region of the acquired spectra are reported.

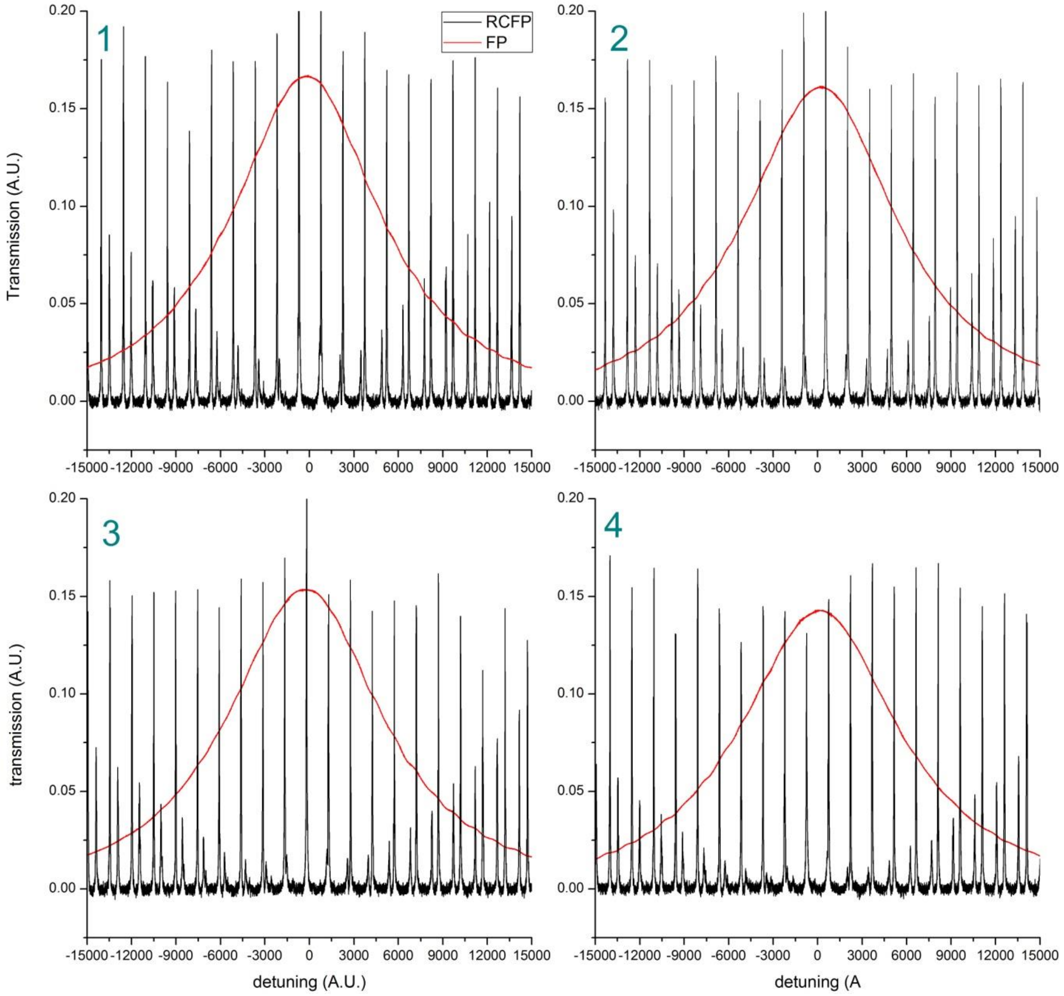

Fig.S3: FP and RCPA spectra (red and black lines), acquired by scanning a narrow-linewidth laser (operating wavelength ~1560nm) across a resonance of the internal FP. The four pairs of spectra shown correspond to 4 different levels of FP losses.

FP resonance: $\boldsymbol{y = y_0 + \frac{2A}{\pi}\frac{w}{4x^2+w^2}}$			
	y0	w	A
1	-0.142	11981.459	76569.234
2	-0.149	12421.831	77028.467
3	-0.155	12561.539	74539.256
4	-0.156	12937.475	71476.272
RCPA dark modes envelope: $\boldsymbol{y = y_0 - A\frac{w}{x^2+w^2}}$			
	y0	w	A
1	0.416	24429.088	9742.841
2	0.323	22608.871	6998.455
3	0.361	28346.453	9989.890
4	0.313	28593.335	8859.598

Table S1: Curve models and best-fit paramenters for the acquired FP resonances and the envelope of the RCPA dark resonances (best fit curves in fig 3a of the main text).

Slope of the absorbance curve (from linear fit)		
Cavity	FP	RCPA
Central	12.732	8.497
Lateral (first 3 points)	3.961	41.153

Table S2: linear-fit slopes for the absorbance measurements plotted in Fig.3b,3c of the main text.
